# Transition from one- to two-dimensional development facilitates maintenance of multicellularity

**DOI:** 10.1098/rsos.160554

**Published:** 2016-09-21

**Authors:** Alejandra M. Manjarrez-Casas, Homayoun C. Bagheri, Akos Dobay

**Affiliations:** Institute of Evolutionary Biology and Environmental Studies, University of Zurich, 8057 Zurich, Switzerland

**Keywords:** generation time, life cycle, evolution of multicellularity, evolution of development, growth in two dimensions

## Abstract

Filamentous organisms represent an example where incomplete separation after cell division underlies the development of multicellular formations. With a view to understanding the evolution of more complex multicellular structures, we explore the transition of multicellular growth from one to two dimensions. We develop a computational model to simulate multicellular development in populations where cells exhibit density-dependent division and death rates. In both the one- and two-dimensional contexts, multicellular formations go through a developmental cycle of growth and subsequent decay. However, the model shows that a transition to a higher dimension increases the size of multicellular formations and facilitates the maintenance of large cell clusters for significantly longer periods of time. We further show that the turnover rate for cell division and death scales with the number of iterations required to reach the stationary multicellular size at equilibrium. Although size and life cycles of multicellular organisms are affected by other environmental and genetic factors, the model presented here evaluates the extent to which the transition of multicellular growth from one to two dimensions contributes to the maintenance of multicellular structures during development.

## 1. Introduction

A key component of multicellular organisms is a developmental life cycle where a multicellular body plan can be constructed and maintained. Such life cycles have evolved several times through the history of life [1–3]. Currently, the term multicellularity applies to a variety of body plans, ranging from filamentous bacteria to higher eukaryotes possessing complex ontogenies [1,3,4]. Multicellular organisms display several properties, which include cell-to-cell adhesion, cell-to-cell communication, multicellular developmental cycle and, in some cases, cell differentiation.

The mechanisms that give rise to multicellular formation are variable, but most fall under two scenarios: (i) a cell division where mother and daughter cells remain together after cell reproduction and (ii) colonial aggregation, where individuals from the same or different species develop certain types of interactions with each other (e.g. cell adhesion, chemotaxis) [1]. The first scenario, where mother and daughter cells fail to separate after division, has played an important role in many of the multicellular transitions known to have happened during the evolution of life. It is, for instance, the basis of all prokaryotic filamentous life forms we know today, and it is likely to have played a basal role in multicellular eukaryotic groups, including plants and animals [1,2].

**Figure 1.**
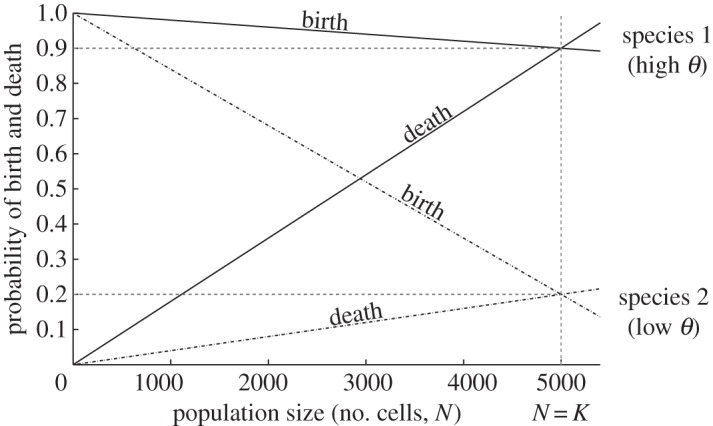
Birth and death rates for two different species as a function of population density (total number of cells at each iteration). The carrying capacity (*K*) is reached when birth and death rates are equal in the population. The rates at carrying capacity are defined here as the turnover rate. The plot shows that the same carrying capacity can be reached by species with different turnover.

The study of cell division and death in a density-dependent context, where mother and daughter cells remain together after division along a single dimension, has provided insights into the variability of the filament sizes that can be observed in strains with the same growth rate (which is equivalent to ‘absolute fitness’ in evolutionary terms) [5]. When birth and death rates are equal at carrying capacity, one can refer to this value as the turnover rate *θ* (see figure 1 and theoretical framework for more details). By looking at various turnover rates, Rossetti *et al.* [5] developed a model based on cell-density-dependent birth and death rates in order to explore filament length distributions. They found that strains with the same growth rate, but different generation time, show different filament length distributions. Hence, a predictable multicellular life cycle can emerge from these scenarios. Their model and the associated experimental evidence facilitate the understanding of simple multicellularity from an ecological perspective. In many cases, variation in generation time can explain the variation of mean filament lengths and the differences in developmental cycles.

Here, we explored the outcome of these dynamics in two dimensions. In particular, we investigated how the transition from one to two dimensions can impact multicellular developmental cycles. This can shed light on the evolution of more complex multicellular structures (when compared to one-dimensional filaments). Our work analyses cell clustering resulting from three main assumptions: (i) an incomplete separation between mother and daughter cells after cell division, (ii) adhesion to adjacent neighbours, and (iii) birth and death rates implemented in a density-dependent manner. Given different birth and death rates, and the previously mentioned assumptions, the population dynamics result in a diversity of cluster sizes that mimic multicellular bodies in two dimensions. The clusters are analysed in terms of their size distribution and maintenance over time. We show that cluster size scales with the turnover rate and discuss its implications on the developmental constraints that can affect the evolution of multicellularity.

## 2. Theoretical framework

Based on a program written in C++, we developed a two-dimensional square lattice model, where each node of the lattice can be either empty or occupied by a cell. Every cell is then subjected to a specified set of rules. Each cell possesses a birth and a death rate that vary in a density-dependent manner. As population density increases, the birth rate decreases, whereas the death rate increases. After a division, mother and daughter cells are not able to separate. Cells adhere to their immediate neighbours if they come from the same cluster. Newborn clusters belonging to the same population grow on independent lattices, eliminating the possibility of cluster re-aggregation. Cell death can lead to the separation of adjacent cells.

### 2.1. Density-dependent birth and death rates

At every iteration, birth and death rates are computed according to the total number of cells present in the population. These are based on the functions $\beta (N_{c})$ for birth and $\delta (N_{c})$ for death rates

2.1 $$\beta (N_{c})=1-c_{1}N_{c}$$

and

2.2 $$\delta (N_{c})=c_{2}N_{c},$$

where $c_{1}=(1-\theta )/K$ and $c_{2}=\theta /K$. *N*_c_ is the total number of cells in the population and *K* is the carrying capacity. *K* is reached when birth and death rates are equal. At carrying capacity, we have β(K)=δ(K)=θ<1, with *θ* being the turnover rate (figure 1). Equations (2.1) and (2.2) are linear with respect to the population size, which is a common assumption used in first-order ecological approximations. In Rossetti *et al.* [5], the authors also explored nonlinear functions (e.g. sigmoidal), but did not observe a significant qualitative change in the results. Based on this experience, we did not try to elaborate on nonlinear functions.

We have further made the simplifying assumption that in two dimensions, cells in the cluster have equal access to a well-mixed nutrient liquid washing over them, as one would observe in a monolayer biofilm. As long as this is the case, a linear function for the density-dependent birth and death rates is a fair approximation, and further geometrical and spatial factors would not have to be considered.

Given equations (2.1) and (2.2), a cell can undergo one of four events during each iteration: divide, die, divide and then die, or take no action. Accordingly, a list containing the events affecting each node is created and its order of execution is randomized. It is also ensured that cells that both divide and die give birth before their death.

### 2.2. Cell division and cluster growth

**Figure 2.**
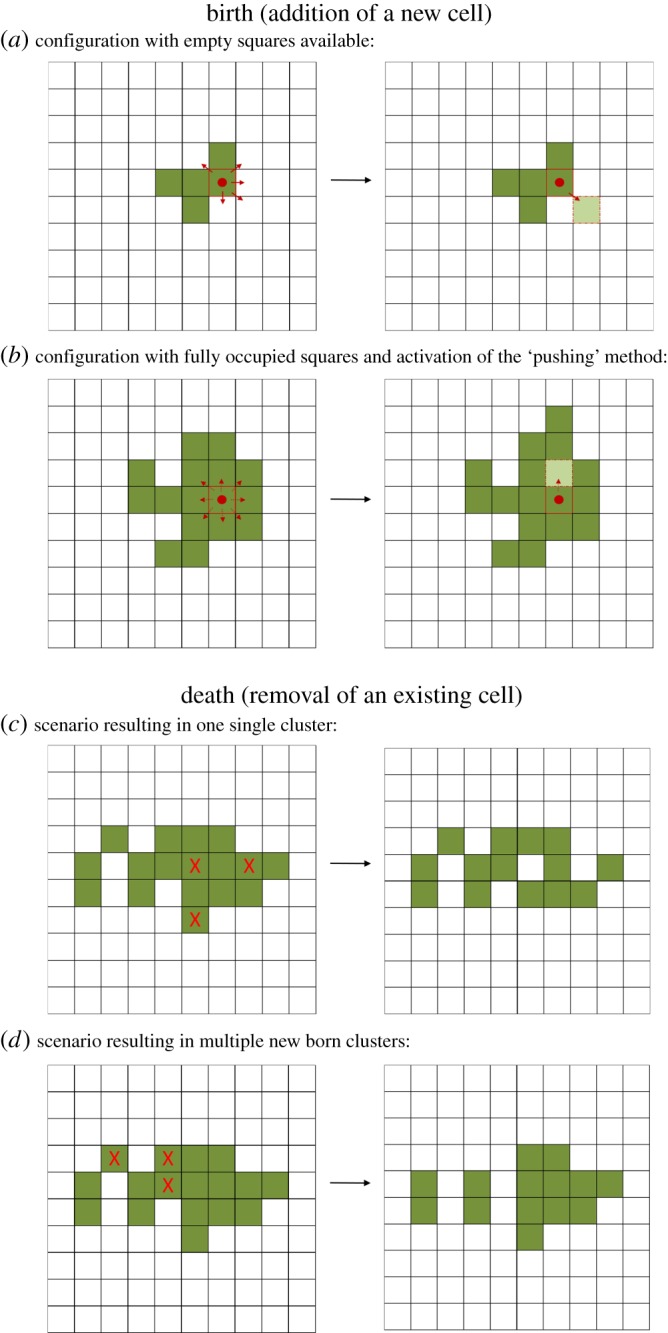
Schematic view representing cluster growth and breakage. (*a*,*b*) Birth: addition of a new cell to the cluster, where the daughter cell is placed next to the mother cell, simulating an incomplete cell division. (*a*) If there are free squares surrounding the mother cell, one of them will be chosen randomly to place the new cell. (*b*) If there are no free squares next to the mother cell, one of the eight squares surrounding the mother cell will be chosen randomly and the daughter cell will be positioned on this square, shifting the previous occupant of the square as well as all the cells adjacent to it. (*c,d*) Removal of cells owing to death can result in cluster breakage, depending on whether the positions occupied by the dead cells represented the only link among two or more cluster sections.

If a cell receives the instruction to divide, then its neighbourhood is evaluated for empty nodes; if there is more than one empty node, then one of them is randomly chosen, and the daughter cell will be placed there (figure 2*a*). In the absence of a free node, an empty position is created to accommodate the daughter cell by shifting a neighbouring node in a random direction. If more than one cell needs to be shifted, then all cells placed along the same direction are moved to their respective neighbour's position along the same direction (figure 2*b*). When a cell dies inside a cluster, the occupied space becomes free (figure 2*c*). Furthermore, every time a cell dies, the cluster to which it belongs is evaluated to determine whether this death induces a cluster break, which results in one or more nascent clusters. A cluster break occurs only on the death of a cell that links two or more regions of the cluster (figure 2*d*). Every nascent cluster is grown on a new lattice in order to avoid interactions that can affect their size and shape. Movie S1 in the electronic supplementary material exemplifies the growth and breakage of the largest cluster in a population.

### 2.3. Neighbourhood and cell adhesion

The neighbourhood of a cell refers to its immediate neighbours separated by a single position. In our two-dimensional lattice model, the neighbourhood of a focal cell corresponds to a total of eight positions, which is known as the Moore's neighbourhood in the field of cellular automata (figure 2). A daughter cell can only occupy positions in the neighbourhood of the mother cell. The model assumes mother–daughter adhesion after cell division. Additionally, while growing in the same cluster, all newly adjacent cells are linked to their neighbour cells.

## 3. Results

**Figure 3.**
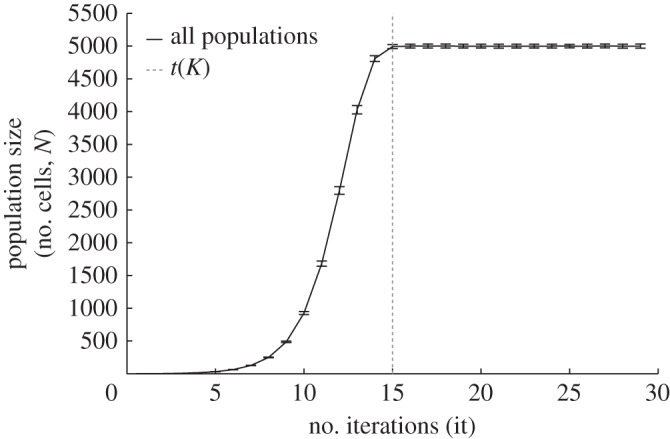
Growth rate across populations in one and two dimensions. The plot shows the mean and standard deviation of the growth curve of all the populations studied: five different turnovers (0.9, 0.5, 0.1, 0.01, 0.001) in both one- and two-dimensional simulations (100 samples each). The dashed line indicates the number of iterations *t*(*K*) at which the carrying capacity is reached. Although each scenario has different birth and death rates and morphology, all populations reach their carrying capacity at the same time, indicating the same growth rate.

We first investigated how the growth rate in one and two dimensions varies with the turnover. In the one-dimensional case, our results were in agreement with Rossetti *et al.* [5]: all populations in one dimension have the same growth rate, independent of their turnover (figure 3). Furthermore, the *in silico* populations showed equal growth rate, whether they were grown in one or two dimensions (figure 3). This first evaluation ruled out the possibility that diversity in multicellular development in these simulations arises from differences in growth rate.

### 3.1. Growth in two dimensions increases cluster size at carrying capacity

**Figure 4.**
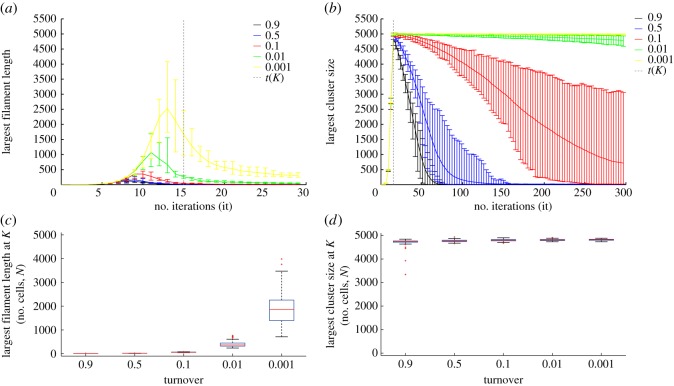
Cluster size at carrying capacity for various turnovers in one and two dimensions. (*a*) The longest filament length (cells) in one dimension and (*b*) the largest cluster size (cells) in two dimensions as a function of the number of iterations. The different colours indicate the different turnovers (from 0.001 in yellow to 0.9 in black), and the dashed line the number of iterations *t*(*K*) at which the carrying capacity is reached (in both cases, this occurs at the 15th iteration, because the growth curve for all turnovers in both dimensions is the same; see also figure 3). In contrast to filaments growing in one dimension, clusters in two dimensions can reach larger sizes and maintain them for a longer time. The central mark shows the mean and the extremes of the error bars represent the upper and lower limits of 95% of the data points. (*c*,*d*) Box plots of the largest filaments and cluster sizes at carrying capacity (at the 15th iteration) for one and two dimensions, respectively. The central bar represents the median, the edges of the box show the 25th and 75th percentiles, the whiskers extend to the extreme data points not considered outliers, and the outliers are plotted individually as red crosses.

Filament lengths resulting from a one-dimensional growth process have been shown to be dependent on the turnover *θ* [5]. Our results corroborated this relation in one dimension and showed that, by decreasing the turnover, the maximum filament length before reaching carrying capacity in each population was increased (figures 4*a,c*). For comparison, we investigated the formation of multicellular clusters in two dimensions. We were interested in understanding the extent to which turnover can affect the cluster sizes in a two-dimensional growth process. We simulated the growth of individual cells that remain together after an incomplete cell division and adhere to their immediate neighbours. The results indicate that the transition of the growth process from one to two dimensions changes the size dynamics of multicellular formation considerably.

First, the turnover did not significantly affect multicellular formation during the exponential growth phase in two dimensions. The largest cluster in every simulated population grew at a similar rate and reached a similar maximum size at carrying capacity, independent of turnover (figure 4*b,d*). In contrast, filaments growing in one dimension showed more turnover-dependent variability during the exponential growth phase (figure 4*a*). In one dimension, the longest filament in each population reached its maximum length after a different number of iterations, depending on the turnover. For instance, the peak of the longest filament appeared later in populations with low turnovers than those with high turnovers, but always before the carrying capacity was reached (figure 4*a*). Moreover, populations with low turnovers, in comparison with high turnovers, displayed longer filaments during the exponential growth phase (figure 4*a,b*). The overall maximum length reached by one-dimensional filaments was around 60% of the number of cells at carrying capacity. This length was only reached for the lowest turnover (*θ* = 0.001). The maximum length in populations with the largest turnover did not reach 4% of the carrying capacity (figure 4*a*). In two dimensions, the maximum size reached by clusters is almost that of the population size at carrying capacity. More than 96% of the cells within the same population belonged to the same cluster at a time point close to the transition from exponential to stationary phase.

These results show that the turnover mainly affects the variability in size of one-dimensional filaments, but not of the two-dimensional clusters (figure 4). The maximum size of the two-dimensional clusters is governed more by the carrying capacity than by the turnover. Furthermore, transition of growth to a two-dimensional plane notably increased the maximum size of multicellular formations.

### 3.2. Growth in two dimensions facilitates the maintenance of large cell clusters

In order to compare how filaments and clusters maintained larger sizes, we defined a *maintenance period*, which is the number of iterations required for a filament or a cluster at its maximum size to break down and reach a *stationary size*.

**Figure 5.**
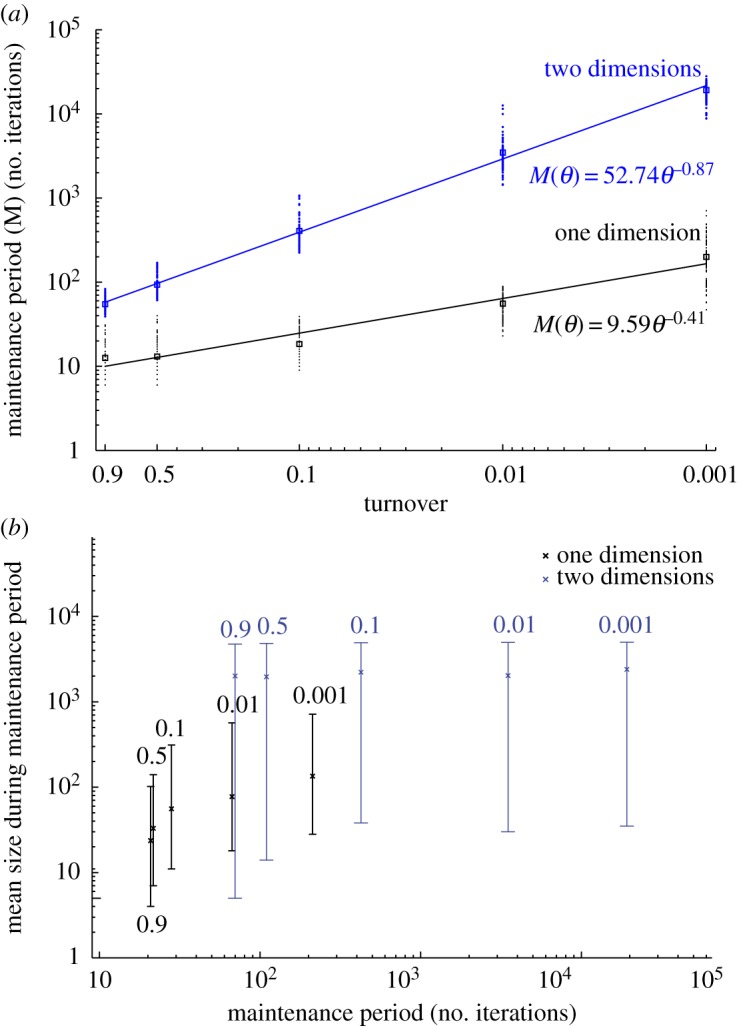
Maintenance period. (*a*) Mean number of iterations during the maintenance period, i.e. time between the iteration when the largest filament or cluster reaches its maximum size and the iteration when it reaches the stationary size. The iterations are plotted as a function of the turnover for one-dimensional (black) and two-dimensional (blue) simulations. The methodology to assess the iteration at which each simulation reaches stationary size is described in the Material and methods section. Each circle represents one run while squares represent the mean value over 100 sets of independent runs for the different turnovers in one and two dimensions. The data can be fitted with a power law (line), with scaling exponents −0.41 (one dimension) and −0.87 (two dimensions). (*b*) Size of the largest filament or cluster during the maintenance period as a function of the length of the maintenance period (number of iterations). The central mark shows the mean and the extremes represent the upper and lower limits of 95% of the data points. The variability of sizes in the graph reflects how the largest filament or cluster changes its size in each population during this period, as it starts with its maximum size and reaches its lowest size (stationary size).

In the one-dimensional scenario, maximum and average filament lengths started decreasing before the end of the exponential phase [5] (figure 4*a*). However, the largest clusters growing in two dimensions took longer to decrease in size and reach their stationary size (four times longer for the highest turnover, and almost 100× longer for the lowest turnover; electronic supplementary material, table S2 and figure 5*a*). We subsequently used a non-parametric hypothesis test (details in Material and methods section) to check if the median and distribution of the number of iterations during maintenance time were significantly different in one- or two-dimensional growth conditions. Results showed that populations with the same turnover maintained the largest cluster size for significantly longer periods of time when they grew in a two- versus one-dimensional environment (electronic supplementary material, table S1). Furthermore, our results showed that the number of iterations during the maintenance period scaled with the turnover (figure 5*a*).

The size of filaments and clusters also differed during the maintenance period, depending mainly on the number of growth dimensions in addition to turnover in the case of one-dimensional filaments (figure 5*b*). The size of the largest clusters in two-dimensional populations did not show any difference among the different turnovers. The average size of the largest clusters in two dimensions exceeded the average size of the largest filaments in one dimension by more than one order of magnitude in all cases (figure 5*b*).

### 3.3. Clusters are further maintained once the stationary size is reached

**Figure 6.**
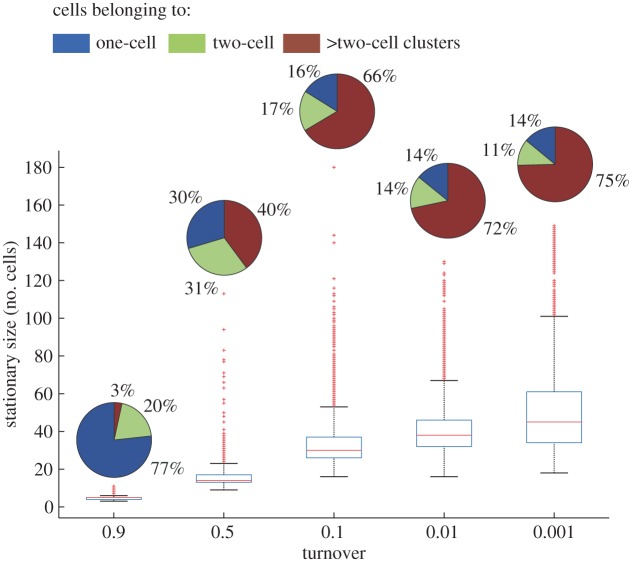
Stationary size in two-dimensional growth. The box plots show the size of the largest cluster per turnover during 100 iterations after stationary size is reached for 100 independent simulation experiments. The central bar represents the median, the edges of the box show the 25th and 75th percentiles, the whiskers cover the extreme data points not yet considered as outliers and the outliers are plotted individually as red crosses. Pie charts illustrate the relative percentage of cells, within a population of 5000 cells at carrying capacity, belonging to one-cell, two-cell or to larger clusters for a given number of iterations, once the stationary size has been reached.

We investigated whether the clusters were preserved or if they dissolved into one-cell elements once the maintenance period was over. We continued to observe variably sized clusters beyond the maintenance period (pie charts in figure 6). The size distribution of the largest clusters in each population was found to be turnover-dependent. The largest clusters generated from the lowest turnover (*θ* = 0.001), for instance, maintained an average size above 50 cells, and up to 150 cells in some cases (boxplots in figure 6). Moreover, we observed that after the largest cluster reached a stationary size in each population, the majority of clusters had at least two cells. The proportion of clusters with more than two cells could reach more than 75% of the total number of clusters present in the population at the lowest turnover (pie charts in figure 6).

## 4. Discussion

Filamentous growth, which represents cell divisions without detachment in one dimension, is a common pattern observed in biological systems. Throughout the evolution of life, the transition of multicellular growth from one to two dimensions allowed access to a new phenotypic morphological space. Two important eukaryotic clades provide examples of this transition. In the plant lineage, once restrictions posed by the cell wall were overcome, body plans changed from an unbranched filamentous organization into a branched and pseudo-parenchymatous morphology [6,7]. Animals, which lacked a cell wall, and consequently the one-dimensional restriction that it posed, did not have to go through a filamentous stage. Hence, they had the potential to develop directly into two- or three-dimensional growth after the transitions from a unicellular to a multicellular state [7].

Filamentous-branched morphologies can be perceived as an intermediate state between filamentous and clustered multicellular organisms (both in two and three dimensions). Compared with three-dimensional clustered structures, filamentous growth and two-dimensional structures offer better access to resources for each individual cell in the structure. On the other hand, the average physical distance between two cells or groups of cells can potentially be reduced in compact three-dimensional architectures in comparison with structures growing in one or two dimensions without subsequent folding. As Schlichting [8] has hypothesized, differences in geometrical placement of cells can lead to differences in the cellular environment. These differences (a kind of symmetry breaking) can subsequently lead to the differences in cellular differentiation states and the evolution of distinct developmental programmes. Hence, moving towards more complex algorithms with differences in individual cellular environments would be a potentially informative extension of this work. Once one allows for differences in cellular environments, and the associated problem of how nutrients are transported and distributed across distances, then one enters the arena of some of the most important problems in the evolution of multicellular development. These include fundamental problems such as division of germline versus soma [9], terminal differentiation [10], vascularization [11] and motility [12].

In this work, we analysed the transition from one to two dimensions and its developmental implications in an ecological context. It has been shown previously that a density-dependent model with the incomplete division assumption permits a multicellular life cycle in one dimension [5]. Here, we tested the effect of a similar model in two dimensions on multicellular organization. We assumed both incomplete cell division and adhesion to close neighbours to mimic events in pseudo-parenchymal growth. Given the same carrying capacity, clusters growing in two dimensions under these assumptions can reach larger sizes in comparison with filaments in one dimension. The most striking difference, however, lies in the maintenance time for the longest one-dimensional filaments and the largest two-dimensional clusters. It takes 4–100 times longer to break two-dimensional clusters to their stationary size in comparison with one-dimensional filaments. This difference is a direct result of having cells in multicellular clusters connected to a larger number of neighbours than those in one-dimensional filaments. Thus, a transition from one to two dimensions results in an increased robustness in organism size maintenance. Moreover, the maintenance period scales inversely with the turnover and exhibits a power law with a scaling exponent circa twofold larger than in one dimension. We hypothesize that in a three-dimensional scenario, where a cell can have up to 26 neighbours, multicellular development would be even more robust as breakage probability decreases with higher number of connected neighbours.

Although size and life cycles of many multicellular organisms can also be affected by other genetic, environmental, ecological and physical factors, this model has allowed us to evaluate, in more general terms, how growth in two dimensions can trigger and support multicellular states. Examples of such ecological and physical factors are, for instance, various shear forces, predator attacks, sharp obstacles, etc. In two dimensions, cells have equal access to the nutrient liquid washing over the cluster. Therefore, local depletion of resources does not represent a strong impediment for evolving multicellular structures as in three dimensions. The results of the model imply the potential importance of density-dependent cell death in multicellular development. In the context of one-dimensional growth, on average, cell death reduces filament length by half [5], as a dying cell can break a filament in two. Cell death in two-dimensional clusters has a lower likelihood of breaking the cluster. However, cell death in a two-dimensional cluster can affect shape. For instance, it has been shown that localized cell death plays a role in biofilm formation [13] and multicellular development in an experimentally evolved yeast [14], complemented with theoretical models [15,16]. Programmed cell death is also known to influence development in animals [17]. If ecological death played a role in the early evolution of multicellularity, then an important question is whether this density-dependent death was subsequently genetically assimilated and evolved into programmed cell death.

Empirical tests can provide more insight on the role of turnover in multicellular development in two or even three dimensions. As genetic programmes in most multicellular organisms play an important role in size and shape, it would be necessary to investigate simple organisms that could allow us to better understand the role of turnover in the early evolution of multicellularity. An interesting alternative would be to study the growth of clusters of cancer cells. It has been previously suggested that cancer cells can give insights into the emergence of multicellularity. For instance, many genes that are associated with the early evolution of metazoans have an important role in the growth of tumours [18,19] and, moreover, some authors have suggested that cancer could putatively be an atavistic state of multicellular life, involving adhering cells with limited cellular cooperation and moderate division of labour [20]. Thus, exploring the role of turnover in tumour cells under carrying capacity could be an experimental platform to test the theoretical work presented here.

## 5. Material and methods

### 5.1. *In silico* model

The two-dimensional square lattice model described in the Theoretical framework section was written in C++ in the form of a command-line executable. Node topology was analysed using the open-source library *LEMON* (Library for Efficient Modelling and Optimization in Networks) [21]. Uniform random numbers were generated using an open-source library in C++ written by Agner Frog that implements an SIMD-oriented fast Mersenne twister algorithm [22].

### 5.2. Definition of duration (number of iterations) and size (number of cells) for the maintenance period

As defined in the Results section, the maintenance period is the number of iterations required for a filament or cluster at its maximum size to break down and reach a stationary size, where variations in size remain within a specific fraction of the maximum size for an indefinite time period. The maintenance period starts when the largest filament or cluster in a population reaches its maximum size and ends at the iteration (*i_*f*_*) when stationary size is reached. In order to find the latter for each turnover, we defined an initial point *i* along the number of iterations axis (the *x*-axis in our case). This initial value *i* was chosen heuristically with the basic requirement of referring to an early iteration point, usually after maximum size is reached, and notably much earlier before the maintenance period is over. Two windows along the same axis were additionally defined: the first window (*w*_1_) goes from iteration 1 to iteration *i* = 50, and the second (*w*_2_) from iteration *i* to iteration 2*i*. We then calculated the range *r*_1_ and *r*_2_ resulting from the size variation over each of the two windows *w*_1_ and *w*_2_, and we repeated this procedure subsequently incrementing the value *i* along the *x*-axis until *i* reaches half of the iterations run for the sample, coinciding with the measurement of the largest windows *w*_1_ and *w*_2_. Note that in some cases, where the stationary size is located after the half of the full data range, the starting value of *w*_1_ was different from iteration 1 and was shifted to the right of the *x*-axis. We then compared the difference obtained for each pair of range values and defined the iteration *i* with the largest positive difference (*r*_1_ − *r*_2_) as the iteration value when stationary size is reached (*i_*f*_*). We repeated the procedure for each simulation in both dimensions and with the five different turnovers.

In order to measure the size during the maintenance period, we counted the number of cells in the longest filament or in the largest cluster over time, starting from the iteration when it reached its maximum size and ending at the iteration when the stationary size was achieved.

### 5.3. Measuring stationary size

The stationary size was defined by taking the mean of the size of the longest filaments or the largest clusters per turnover for 10 (one dimension) or 100 (two dimensions) iterations after leaving the maintenance period. This difference in the total number of iterations defining the value of the stationary size for both dimensions depended on the number of iterations of our dataset.

Additionally, a subset of all clusters that persist after the maintenance period was chosen to determine the relative percentage of cells in the populations of each turnover in two dimensions that belong to one-cell, two-cell or larger clusters.

### 5.4. Statistical test

In order to evaluate the different lengths of the maintenance period, we performed a pairwise comparison of one- and two-dimensional turnover populations generated at equal turnover rates. Because the data were not assumed to be normally distributed, a Wilcoxon rank-sum test was applied as a non-parametric hypothesis. For each tested pair, the null hypothesis of having an equal median of number of iterations was rejected (electronic supplementary material, table S1). The Wilcoxon rank-sum test was performed using Matlab.

## Supplementary Material

Turnover rates in one and two dimensions

## Data accessibility

The datasets used in this study are available at Dryad under accession number at http://dx.doi.org/10.5061/dryad.bj0b2 [23]. The associated code for fitting and simulating the model is also available upon request.

## References

[1] Bonner JT 1998 The origins of multicellularity. Integr. Biol. 1, 27–36. (doi:10.1002/(SICI)1520-6602(1998)1:1<27::AID-INBI4>3.0.CO;2-6)

[2] Rokas A 2008 The molecular origins of multicellular transitions. Curr. Opin. Genet. Dev. 18, 472–478. (doi:10.1016/j.gde.2008.09.004) 18926910 10.1016/j.gde.2008.09.004

[3] Grosberg RK, Strathmann RR 2007 The evolution of multicellularity: a minor major transition? Annu. Rev. Ecol. Evol. System. 38, 621–654. (doi:10.1146/annurev.ecolsys.36.102403.114735)

[4] Resendes M, António DS, Schulze-Makuch D 2012 Toward a new understanding of multicellularity 2. Evidence for an evolutionary path to multicellularity. Hypotheses Life Sci. 2, 4–14.

[5] Rossetti V, Filippini M, Svercel M, Barbour AD, Bagheri HC 2011 Emergent multicellular life cycles in filamentous bacteria owing to density-dependent population dynamics. J. R. Soc. Interface 8, 1772–1784. (doi:10.1098/rsif.2011.0102) 21593029 10.1098/rsif.2011.0102 PMC3203479

[6] Niklas K 2000 The evolution of plant body plans—a biomechanical perspective. Ann. Bot. 85, 411–438. (doi:10.1006/anbo.1999.1100)

[7] Niklas KJ, Newman SA 2013 The origins of multicellular organisms. Evol. Dev. 15, 41–52. (doi:10.1111/ede.12013) 23331916 10.1111/ede.12013

[8] Schlichting CD 2003 Origins of differentiation via phenotypic plasticity. Evol. Dev. 5, 98–105. (doi:10.1046/j.1525-142X.2003.03015.x) 12492416 10.1046/j.1525-142x.2003.03015.x

[9] Buss L 1987 The Evolution of individuality. Princeton, NJ: Princeton University Press.

[10] Matias Rodrigues JF, Rankin DJ, Rossetti V, Wagner A, Bagheri HC 2012 Differences in cell division rates drive the evolution of terminal differentiation in microbes. PLoS Comput. Biol. 8, e1002468 (doi:10.1371/journal.pcbi.1002468) 22511858 10.1371/journal.pcbi.1002468 PMC3325182

[11] Chase J 1982 The evolution of retinal vascularization in mammals. Ophthalmology 89, 1518–1525. (doi:10.1016/S0161-6420(82)34608-4) 7162797 10.1016/s0161-6420(82)34608-4

[12] Solari CA, Michod RE, Goldstein RE 2008 *Volvox barberi*, the fastest swimmer of the Volvocales (Chlorophyceae) 1. J. Phycol. 44, 1395–1398. (doi:10.1111/j.1529-8817.2008.00603.x) 27039854 10.1111/j.1529-8817.2008.00603.x

[13] Asally M et al. 2012 Localized cell death focuses mechanical forces during 3D patterning in a biofilm. Proc. Natl Acad. Sci. USA 109, 18 891–18 896. (doi:10.1073/pnas.1212429109) 23012477 10.1073/pnas.1212429109 PMC3503208

[14] Ratcliff WC, Denison RF, Borrello M, Travisano M 2011 Experimental evolution of multicellularity. Proc. Natl Acad. Sci. USA 109, 1595–1600. (doi:10.1073/pnas.1115323109) 22307617 10.1073/pnas.1115323109 PMC3277146

[15] Libby E, Ratcliff W, Travisano M, Kerr B 2014 Geometry shapes evolution of early multicellularity. PLoS Comput. Biol. 10, 15–19. (doi:10.1371/journal.pcbi.1003803) 10.1371/journal.pcbi.1003803 PMC4168977 25233196

[16] Duran-Nebreda S, Sole R 2015 Emergence of multicellularity in a model of cell growth, death and aggregation under size-dependent selection. J. R. Soc. Interface 12, 20140982 (doi:10.1098/rsif.2014.0982) 25551152 10.1098/rsif.2014.0982 PMC4277087

[17] Fuchs Y, Steller H 2011 Programmed cell death in animal development and disease. Cell 147, 742–758. (doi:10.1016/j.cell.2011.10.033) 22078876 10.1016/j.cell.2011.10.033 PMC4511103

[18] Srivastava M et al. 2010 The *Amphimedon queenslandica* genome and the evolution of animal complexity. Nature 466, 720–726. (doi:10.1038/nature09201) 20686567 10.1038/nature09201 PMC3130542

[19] Domazet-Loso T, Tautz D 2010 Phylostratigraphic tracking of cancer genes suggests a link to the emergence of multicellularity in metazoa. BMC Biol. 8, 66 (doi:10.1186/1741-7007-8-66) 20492640 10.1186/1741-7007-8-66 PMC2880965

[20] Davies PCW, Lineweaver CH 2011 Cancer tumors as metazoa 1.0: tapping genes of ancient ancestors. Phys. Biol. 8, 015001 (doi:10.1088/1478-3975/8/1/015001) 21301065 10.1088/1478-3975/8/1/015001 PMC3148211

[21] Dezső B, Jüttner A, Kovács P 2011 LEMON – an open source C++ graph template library. Electron. Notes Theor. Comput. Sci. 264, 23–45. (doi:10.1016/j.entcs.2011.06.003)

[22] Matsumoto M, Nishimura T 1998 Mersenne twister: a 623-dimensionally equidistributed uniform pseudo-random number generator. ACM Trans. Model. Comput. Simul. 8, 3–30. (doi:10.1145/272991.272995)

[23] Manjarrez-Casas AM, Bagheri HC, Dobay A 2016 Data from: Transition from one- to two-dimensional development facilitates maintenance of multicellularity. Dryad Digital Repository. (doi:10.5061/dryad.bj0b2) 10.1098/rsos.160554 PMC5043334 27703714

