# Supplementary material for: Transition from one- to two-dimensional development facilitates maintenance of multicellularity
Source: R Soc Open Sci. 2016 Sep 21;3(9):160554. doi: 10.1098/rsos.160554 (PMC5043334; doi:10.1098/rsos.160554)
Supplement: Turnover rates in one and two dimensions [file rsos160554supp1.pdf]

Supplementary Information

Table S1. Wilcoxon rank sum test performed in order to compare the length of the maintenance period (number of iterations) in populations with the same turnover, but growing in different dimensions. All comparisons showed significant difference.

Turnover compared in 1D versus 2D	P-value
0.9	<0.001
0.5	<0.001
0.1	<0.001
0.01	<0.001
0.001	<0.001

Table S2. Comparison of the longest filament and the largest cluster based on the turnover rate θ and the number of dimensions where the populations are grown.

Turnover rate θ	Longest filament (1D) and largest cluster size (2D) at its maximum size (mean number of cells)		Mean number of iterations to reach the maximum size of the longest filament (1D) and largest cluster (2D)		Stationary size of the longest filament (1D) and largest cluster (2D) (mean number of cells)		Maintenance Period (mean number of iterations)	
	**1D**	**2D**	**1D**	**2D**	**1D***	**2D**	**1D**	**2D**
**0.9**	137.3	4837.5	9.1	16.1	4.03	4.90	12.57	54.73
**0.5**	189.8	4957.8	9.7	17.2	8.29	15.17	13.07	93.56
**0.1**	406.8	5014.5	10.5	18.6	12.71	32.75	18.40	406.43
**0.01**	1146.2	5016.1	12.3	24.6	19.23	40.97	55.63	3470.59
**0.001**	2763.9	5007.6	13.7	27.8	48.24	52.88	199.60	19177.97

*10 iterations after end of maintenance period (in comparison to 100 for 2D)
